# Supplementary material for: Frailty Risk Prediction Model among Older Adults: A Chinese Nation-Wide Cross-Sectional Study
Source: Int J Environ Res Public Health. 2022 Jul 9;19(14):8410. doi: 10.3390/ijerph19148410 (PMC9322778; doi:10.3390/ijerph19148410)
Supplement: Supplementary file 1 [file ijerph-19-08410-s001.zip › ijerph-1758533-SM.pdf]

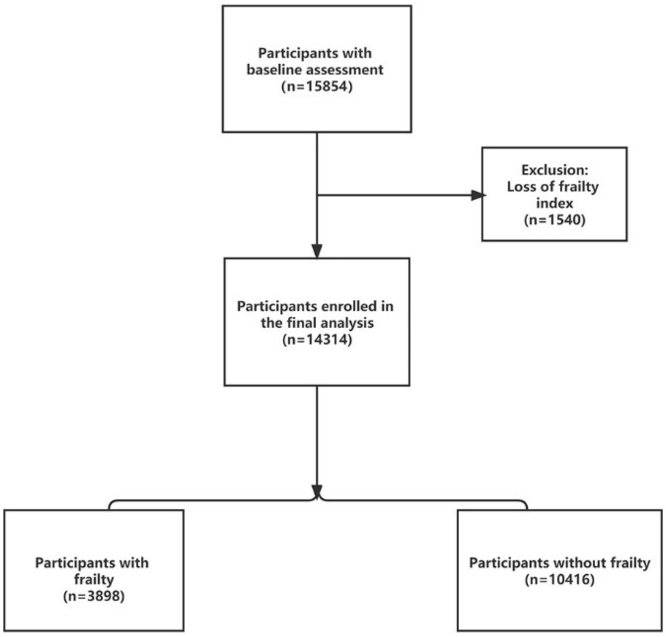

Supplemental Figure S1. The flow chart of participants.

Supplemental Table S1. Detailed information on behavioural and social support factors.

Characteristics	Description
Behavioral Factors (n=35)	
Co-residence	1.With household member(s)<br>2.Alone<br>3.In an institution
Staple food	1.Rice<br>2.Corn (maize)<br>3.Wheat (noodles and bread etc.)<br>4.Half rice and half flour<br>5.Others
Amount of staple food per day	1.<0.2<br>2.0.2-0.5<br>3.>0.5
Edible oil	1.Other vegetable oils<br>2.Gingili grease<br>3.Lard<br>4.Other animal's fat
Main dietary flavour	1.Insipidity<br>2.Salty<br>3.Sweet<br>4.Hot<br>5.Crude<br>6.Others
Drinking water	1.From a well

Characteristics	Description
	2.From a river or lake<br>3.From a spring<br>4.From a pond or pool<br>5.Tap water
Smoking	1.Yes<br>2.No
Drinking	
Exercise	
Having taken a nutrient supplement/ medicine in the past 24 hours	
Times of brushing teeth	1.Do not brush<br>2.Occasionally<br>3.Once<br>4.Twice<br>5.Three or more times
Frequency of eating fresh fruit/ vegetable/ meat/ fish/ egg/ legume/ food/ pickle/ sugar/ garlic/ dairy/ nut/ mushroom or algae/ vitamin/ medicinal plant/ tea	1.Almost every day<br>2.Occasionally<br>3.Rarely or never
Taking protein/ calcium/ iron/ zinc/ compound vitamin/ vitamin A or D/ DHA/ others usually	1.Yes<br>2.No
Social support factors (n=25)	
Number of children	1.0<br>2.1<br>3.2-4<br>4.5-7<br>5.≥8
The first person you want to share thoughts with	1.Spouse<br>2.Children, sons in law or daughters in law<br>3.Others<br>4.Nobody
The first person you ask for help when you have problems	
Primary caregiver when ill	
Primary payer of medical expenses	
Distance from home to the nearest hospital (in kilometers)	1.≤1<br>2.1-2<br>3.2-10<br>4.>10
Able to get adequate medical service	1.Yes<br>2.No
Regular physical examination once every year	
Having any social security and social insurance (retirement pension/ public old-age insurance/ private or commercial old-age insurance/ public free medical services/ urban employee medical insurance/ urban resident medical insurance/ new rural cooperative medical insurance/ commercial medical insurance)	
Having available community service (personal care/ home visit/ spiritual comfort	

Characteristics	Description
and chat/ daily shopping/ social and recreational activity/ legal aid/ health education/ neighborhood-relation/ others).	

Supplemental Table S2. The points for characteristics.

Characteristics	Values	Points	Characteristics	Values	Points
Age (year)			Frequency of taking vegetables		
50-69	0	0	Almost everyday	0	0
70-99	1	49	Occasionally	1	9
≥100	2	99	Rarely or never	2	37
Nationality			Frequency of taking egg		
Han	0	15	Almost everyday	0	7
Minority	1	0	Occasionally	1	0
Residence			Rarely or never	2	12
Urban	0	6	Frequency of taking garlic		
Rural	1	0	Almost everyday	0	0
Education (year)			Occasionally	1	1
0	0	18	Rarely or never	2	10
1-6	1	11	Frequency of taking dairy		
>6	2	0	Almost everyday	0	21
Marital status			Occasionally	1	5
Currently married and living with spouse	0	6	Rarely or never	2	0
Separated	1	18	Frequency of taking nut		
Divorced	2	17	Almost everyday	0	0
Widowed	3	18	Occasionally	1	4
Never married	4	0	Rarely or never	2	20
Main occupation before age 60			Frequency of taking tea		
Professionals and technician	0	29	Almost everyday	0	0
Administrative manager	1	34	Occasionally	1	5
Clerk	2	12	Rarely or never	2	11
Self-employed	3	7	Main source of water		
Agriculture, husbandry, fishery	4	0	From a well	0	12
House worker	5	13	From a river or lake	1	19
Soldier	6	38	From a spring	2	12
Never worked	7	15	From a pond or pool	3	0
Others	8	5	Tap water	4	21
Main source of financial support			Current smoking		
Retirement wages	0	100	Yes	0	0
Relative (s)	1	90	No	1	24
Local government or community	2	85	Current drinking		
Work by self	3	0	Yes	0	0
Self-assessed sufficient economic support			No	1	20
Yes	0	0	Current exercise		

Characteristics	Values	Points	Characteristics	Values	Points
No	1	16	Yes	0	0
Self-rated economic level			No	1	64
Very rich	0	6	Times of brushing teeth everyday		
Rich	1	0	Do not brush	0	43
So so	2	10	Occasionally	1	29
Poor	3	31	Once	2	14
Very poor	4	44	Twice	3	0
Co-residence			Three or more times	4	15
With household member (s)	0	43	Regular physical examination once a year		
Alone	1	0	Yes	0	0
In an institution	2	83	No	1	25
Staple food			First person you want to share thoughts with		
Rice	0	0	Spouse	0	0
Corn (maize)	1	22	Children, sons in law or daughters in law	1	22
Wheat (noodles and bread etc.)	2	12	Others	2	18
Half rice and half flour	3	17	Nobody	3	29
Others		25	Primary caregiver when ill		
Amount of staple food per day (kg)			Spouse	0	61
<0.2	0	23	Children, sons in law or daughters in law	1	68
0.2-0.5	1	6	Others	2	80
>0.5	2	0	Nobody	3	0
Edible oil			Primary payer of medical expenses		
Other vegetable oils	0	15	Urban employee/ resident medical insurance	0	22
Gingili grease	1	20	Cooperative medical scheme	1	12
Lard	2	5	Private medical insurance	2	6
Other animal's fat	3	0	Self	3	6
Main dietary flavor			Spouse	4	29
Insipidity	0	18	Children	5	26
Salty	1	16	No money to pay	6	0
Sweet	2	21	Others	7	28
Hot	3	0	Get adequate medical service		
Crude	4	35	Yes	0	0
Others	5	22	No	1	16
